# α-Solanine Causes Cellular Dysfunction of Human Trophoblast Cells via Apoptosis and Autophagy

**DOI:** 10.3390/toxins13010067

**Published:** 2021-01-18

**Authors:** Zhilong Chen, Chen Li, Anwen Yuan, Ting Gu, Feng Zhang, Xiujun Fan, Xiaosong Wu, Xingyao Xiong, Qing Yang

**Affiliations:** 1 College of Veterinary Medicine, Hunan Agricultural University, Changsha 410128, China; zlongchen@stu.hunau.edu.cn (Z.C.); lichen.0904@foxmail.com (C.L.); yuananweny@hunau.edu.cn (A.Y.); gut9592@stu.hunau.edu.cn (T.G.); zhangfeng0220@foxmail.com (F.Z.); wuxiaosong@hunau.edu.cn (X.W.); 2 Center for Energy Metabolism and Reproduction, Institute of Biomedicine and Biotechnology, Shenzhen Institute of Advanced Technology, Chinese Academy of Sciences, Shenzhen 518055, China; xj.fan@telomerescitech.com; 3 Shenzhen Agricultural Genome Research Institute, Chinese Academy of Agricultural Sciences, Beijing 100081, China

**Keywords:** α-solanine, trophoblast cell, toxicity, apoptosis, autophagy

## Abstract

The trophoblast, an embryonic tissue, exerts a crucial role in the processes of implantation and placentation. Toxins in food can cause malfunction of trophoblasts, resulting in apoptosis, oxidative stress, and abnormal angiogenesis. α-solanine, a steroidal glycoalkaloid, has antitumor properties on several cancer cells. However, its effect on human trophoblasts has not been elucidated. In this study, human extravillous trophoblast HTR-8/SVneo cells were exposed to α-solanine. Cellular functions including proliferation, migration, invasion, tube formation, and apoptosis were assessed. To monitor autophagic flux, trophoblasts were transfected with a mCherry-GFP-LC3B vector using lentiviral transduction, and expression of autophagy-related biomarkers including Beclin 1, Atgl3, and microtubule-associated protein 1 light chain-3 (MAP1-LC3) were detected. The results show that application of 20 μM α-solanine or above inhibited the cell viability, migration, invasion, and tube formation of the human trophoblast. Cell cycle was arrested at S and G2/M phases in response to 30 μM α-solanine. α-solanine induced apoptosis of HTR-8/SVneo cells and triggered autophagy by increasing the autophagic gene expression and stimulating the formation of autophagosome and autophagic flux. In conclusion, α-solanine can impair the functions of human trophoblast cells via activation of cell apoptosis and autophagy.

## 1. Introduction

Potato, rich in carbohydrates, cellulose, proteins, and minerals with low fat content, plays an indispensable role in human diet. Glycoalkaloids (GAs) generated from potatoes and all nightshade plants are natural toxins to protect plants against environmental attacks from insects, bacteria, and animals [1]. α-solanine is one of the main GAs and abundant in potato buds and green peels. It has been revealed that α-solanine possesses potential chemotherapeutic action against cancers in several organs, including lung [2], breast [3], esophagus [4,5], prostate [6,7], and liver [8,9], as well as acute lymphocytic leukemia [10]. α-solanine also has potential anti-inflammatory properties [11,12]. However, potato tubers can accumulate a high concentration of GAs without proper storage, leading to toxicity on human health [13]. A small amount of α-solanine can cause common symptoms such as dizziness, nausea, abdominal pain, vomiting, and diarrhea. Excessive consumption of α-solanine can lead to convulsions, coma, and even death [14]. 

Animal studies have shown that α-solanine can damage the reproductive system. It inhibits the proliferation of mouse sertoli and Leydig cells, leading to toxic effects in themale reproductive system [15]. It induces abortion in mice [16] and damages the embryo and fetus of rats and chickens [17,18]. Embryo development is inhibited when exposure to the steroidal GAs from potatoes in vitro [19]. It also toxically impaired the oocyte maturation, and then suppressed the embryonic development in a pig model [20]. Trophoblast, a peripheral part of blastocyst, plays important roles in implantation and placentation. During human placentation, trophoblast cells invade into the myometrium along with the uterine vasculature. Extravillous cytotrophoblast cells are highly invasive, which ensure the uterine spiral arteries remodeling. Different from tumor cells, the invasion of trophoblasts is a normal physiological behavior with moderation, which is strictly regulated by the mother [21]. With exposure to environmental changes or toxic threats, trophoblast cells become round or detached, and cell abilities such as movement, invasion, and hormone secretion may be altered [22,23,24]. Malfunctioning of trophoblasts leads to inflammation [25], oxidative stress [26,27], apoptosis [28,29], and dysregulated angiogenesis [30]. The toxicity of α-solanine is well-known; however, its toxic effect on human trophoblasts is not yet understood. 

In this study, we investigated the effect of α-solanine on cell functions of human trophoblast in vitro. To determine the potential molecular mechanism of α-solanine, we explored the changes in apoptosis, autophagic protein expression, and formation of autophagosome and autophagic flux in the trophoblast cells.

## 2. Results

### 2.1. α-Solanine Inhibited Proliferation and Induced Cell Cycle Arrest of HTR-8/SVneo Cells

**Figure 1:**
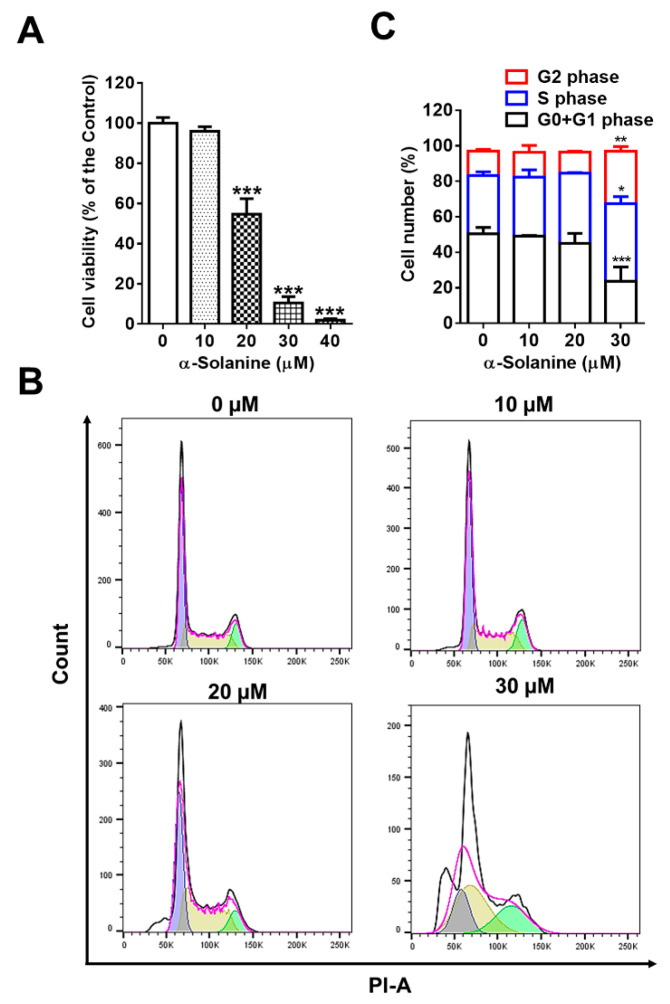
α-solanine inhibited cell proliferation and induced cell cycle arrest in HTR-8/SVneo cells. (**A**) Cell viability was determined by CCK8 assay after exposure to α-solanine (0, 10, 20, 30, and 40 µM) for 24 h, six wells per dose. (**B**,**C**) Cell cycle distribution of HTR-8/SVneo cells treated with α-solanine (0, 10, 20, and 30 µM) for 24 h was measured by flow cytometry analysis in triplicate. Values are the means ± S.D. (*n* = 3). * *p* < 0.05, ** *p* < 0.01, *** *p* < 0.001 indicate statistically significant difference compared with the control.

The effect of α-solanine on proliferation of HTR-8/SVneo cells was evaluated. As shown in Figure 1, α-solanine inhibited cell proliferation of HTR-8/SVneo when cells were treated with doses of 20 μM or above (Figure 1A). Cell cycle was analyzed using flow cytometry assay. The results show that 30 μM of α-solanine treatment significantly increased the cell populations of S and G2 phases, and 20 μM α-solanine induced S-phase cell cycle arrest (Figure 1B,C).

### 2.2. α-Solanine Induced Apoptosis in HTR-8/SVneo Cells

**Figure 2:**
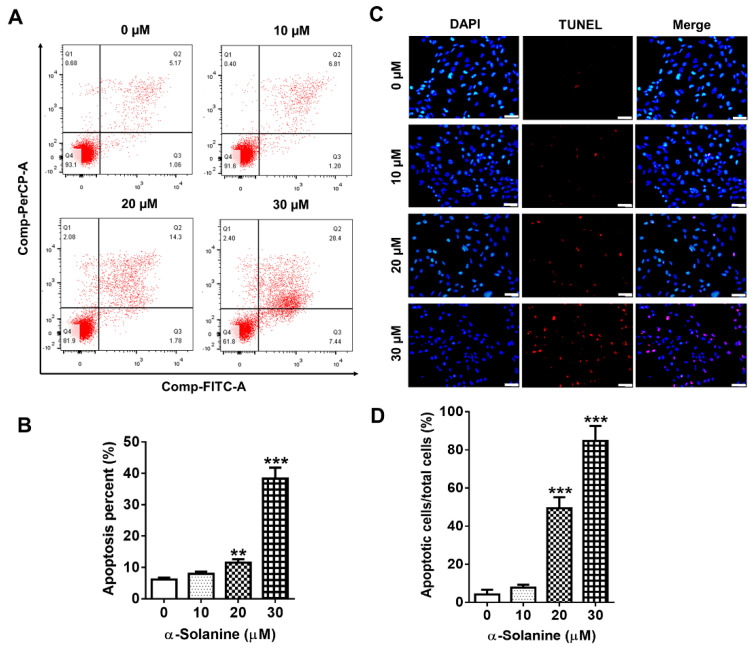
α-solanine induced cell apoptosis of HTR-8/SVneo. Cells were treated with 0, 10, 20, and 30 μM α-solanine for 18 h: (**A**) representative images from flow cytometry analysis with fluorescein isothiocyanate (FITC)-labeled Annexin V and propidium iodide (PI) double staining; (**B**) the analysis of the percentage of apoptotic cells; (**C**) representative images of apoptotic cells from TUNEL assay in situ (scale bar = 40 µm); and (**D**) quantification and analysis of the apoptotic cells. Data are presented as means ± S.D. (*n* = 3). ** *p* < 0.01, *** *p* < 0.001 indicate statistically significant difference compared with the control.

To explore whether the cytotoxic effect of α-solanine was related to cell apoptosis, HTR-8/SVneo cells were exposed with different concentrations of α-solanine. As shown in Figure 2A,B, the percentages of apoptotic cells including the early apoptotic cells in quadrant 2 (Q2) and late apoptotic cells in Q4 were significantly increased in response to α-solanine at 20 (11.5%, *p* < 0.01) and 30 μM (38.3%, *p* < 0.001) compared to the control (6.14%). TUNEL staining was performed to confirm the apoptotic activity of α-solanine in HTR-8/SVneo cells, which also showed that the number of apoptotic cells increased when treated with higher concentrations (20 and 30 μM) of α-solanine, while doses of 10 μM or below had no significant effect on cell apoptosis (Figure 2C,D).

### 2.3. α-Solanine Inhibited Invasion and Migration of HTR-8/SVneo Cells

**Figure 3:**
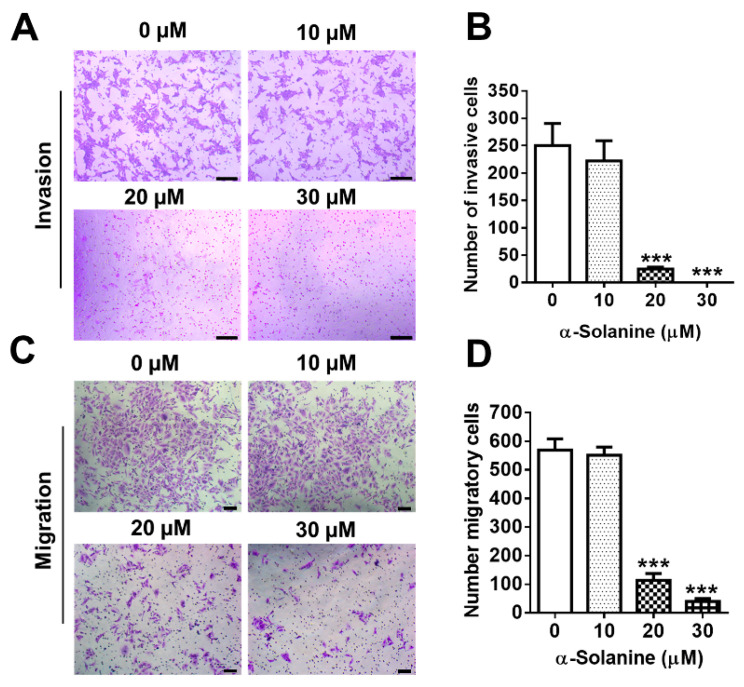
α-solanine inhibited trophoblast invasion and migration. The invasion and migration of cells were measured by transwell insert with or without Matrigel-coated polycarbonate filters (8 µm pore size) in the presence of α-solanine (0, 10, 20, and 30 μM) for 18 h, respectively. (**A**) Representative images of the cells across the filters (scale bar = 100 µm). (**B**) The invaded cells were counted from six random fields. (**C**) Representative images of the cells migrating to the lower side of the filters (scale bar = 100 µm). (**D**) The migratory cells were counted from six random fields. Data are presented as means ± S.D. (*n* = 3). *** *p* < 0.001 indicates significance compared with the control.

Trophoblast invasive ability was determined by Matrigel transwell invasion assay. A significant decrease in the number of cells invading through transwell membrane coated with Matrigel was observed when HTR-8/SVneo cells were exposed to 20 μM α-solanine (*p* < 0.001); few cells invaded through the membranes when exposed to 30 μM α-solanine; and 10 μM α-solanine showed no effect on cell invasion (Figure 3A,B). The migration assay showed similar results (Figure 3C,D).

### 2.4. α-Solanine Inhibited the Endothelial-Like Tube Formation in HTR-8/SVneo Cells

**Figure 4:**
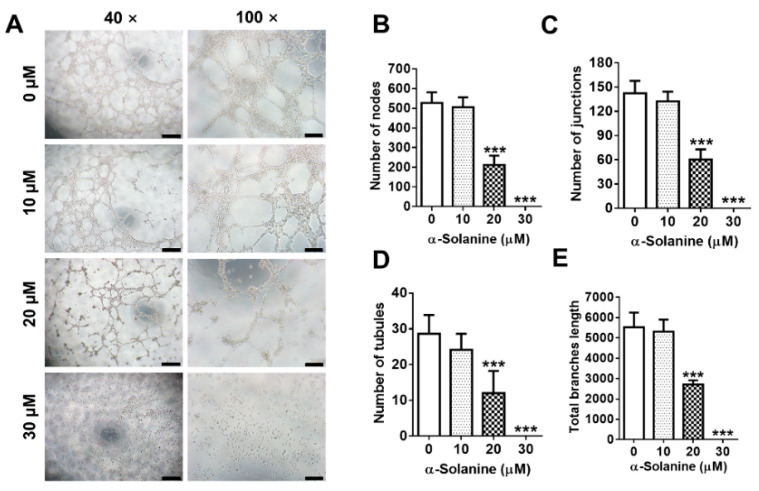
α-solanine inhibited the tube formation of HTR-8/SVneo cells. Tube formation was detected after the treatment of HTR-8/SVneo cells with α-solanine (0, 10, 20, and 30 μM) for 6 h, respectively. (**A**) Representative images from the endothelial-like tube formation (left scale bar = 2 mm, right scale bar =100 µm). (**B**–**E**) The number of nodes, junctions, and tubules and the total branches length were quantitated from four images at 100× magnification, respectively. Data are presented as means ± S.D. (*n* = 3). *** *p* < 0.001 indicates significance compared with the control.

HTR-8/SVneo cells have an endothelial cell-like behavior to form networks [31]. We investigated the regulation of α-solanine on the endothelial-like tube formations of HTR-8/SVneo cells. Treatment with 10 μM α-solanine showed no change in the number of nodes (Figure 4A,B), junctions (Figure 4A,C), and tubules (Figure 4A,D), as well as the total branches length (Figure 4A,E). The ability of endothelial-like tube formation decreased significantly when exposed to 20 μM α-solanine, and no tube formation was observed when HTR-8/SVneo cells were treated with 30 μM of α-solanine.

### 2.5. α-Solanine Triggered Autophagy in HTR-8/SVneo Cells

**Figure 5:**
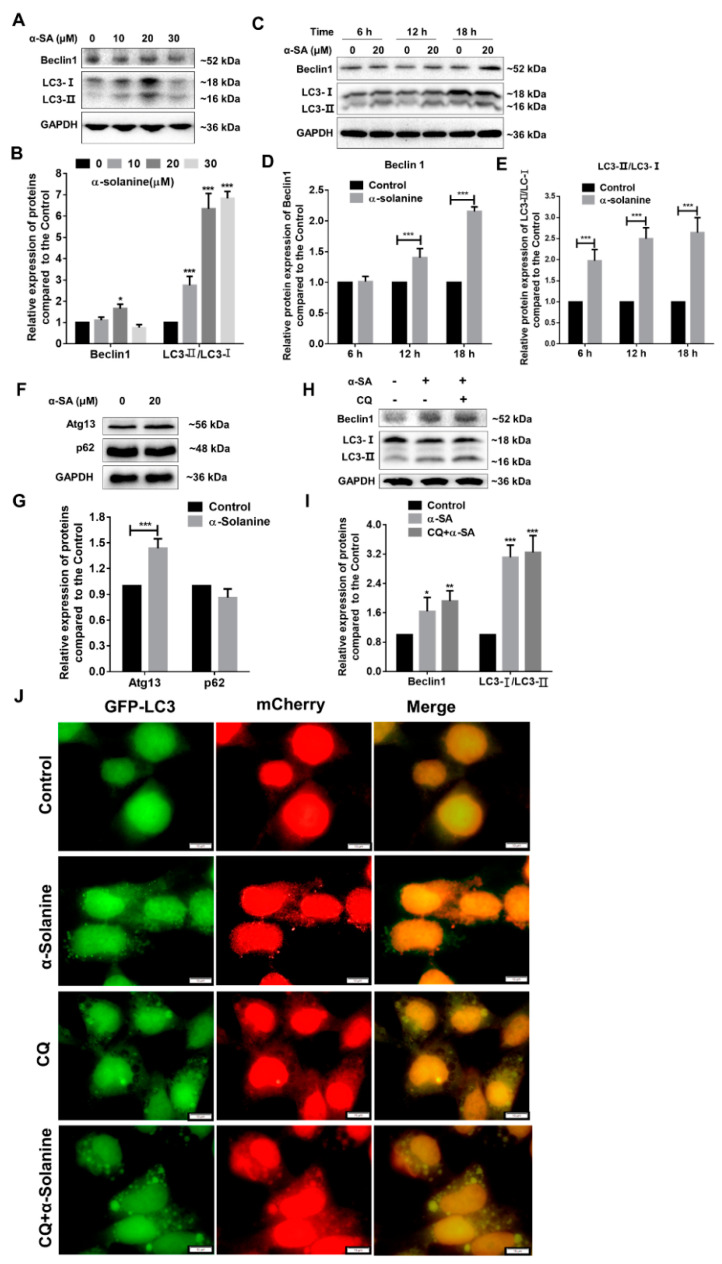
α-solanine induced autophagy in HTR-8/SVneo cells. (**A**,**B**) Trophoblasts were incubated with the indicated doses of α-solanine (0, 10, 20, and 30 μM) for 18 h, and the expressions of Belclin 1 and LC3 were detected by Western blot. (**C**–**E**) Cells were exposed to 20 μM α-solanine for 6, 12, and 18 h, and the expressions of Belclin 1 and LC3 were analyzed. (**F**,**G**) Cells were treated with 20 μM α-solanine for 18 h, and autophagic pathway proteins Atg13 and p62 were analyzed. (**H**,**I**) Cells were co-treated with 20 μM α-solanine and 50 µM chloroquine (CQ) for 18 h, and the expression of Belclin 1 and LC3 were analyzed. (**J**) HTR-8/SVneo cells were stable transfected with lentivirus-mediated mCherry-GFP-LC3B vector and treated with 20 µM α-solanine or co-treatment with 50 µM CQ for 18 h. The fluorescence signals were visualized and photographed under a fluorescent microscopy (scale bar =10 µm). The yellow puncta represent autophagosome, while the red puncta represent autolysosomes. Relative intensity of the indicated protein expression was calculated by ImageJ and normalized to control. GAPDH was used as an internal control. Values are presented as means ± S.D. (*n* = 3); * *p* < 0.05, ** *p* < 0.01, *** *p* < 0.001 indicate significant difference compared to control.

To investigate the effect of α-solanine on autophagy of HTR-8/SVneo, cells were treated with different concentrations of α-solanine. The results from immunoblotting show that α-solanine increased significantly the ratio of LC3I/LC3 II, which peaked with 20 µM α-solanine treatment (Figure 5A,B,E and Figure S1). Autophagic markers, Beclin 1, and autophagy-regulated 13 (Atg13) levels was also increased when cells were exposed to 20 µM α-solanine, but p62 expression showed no change (Figure 5A,B,F,G). These results indicate that autophagy is activated in HTR-8/SVneo cells after α-solanine treatment. To verify the activation of autophagy, trophoblast cells were co-treated with chloroquine (CQ), a common inhibitor of autophagy. The results show that co-treatment with CQ and α-solanine increased the Beclin 1 level as well the ratio of LC3 II/I (Figure 5H,I). A tandem fluorescent LC3 (mCherry-GFP-LC3B) expression vector was used to further monitor the fusion of autophagosome and lysosome. As shown in Figure 5J, the number of green and red fluorescent puncta were increased in cells after α-solanine treatment when compared to the controls; co-treatment with α-solanine and CQ increased the yellow LC3 puncta. The results further confirm that α-solanine could induce the formation of autophagosome and promote the autophagic flux in human trophoblast cells.

## 3. Discussion

The present findings illustrate the α-solanine-induced toxic effects on human trophoblast cells, which inhibited cell proliferation and induced cell cycle arrest. Moreover, α-solanine suppressed the ability of tube formation, cell invasion, and migration when trophoblast cells were treated with 20 μM or above. 

HTR-8/SVneo, a human trophoblast cell line, derived from the first trimester extravillous trophoblast infected with SV40 large T antigen, is a widely used model of extravillous trophoblast [32,33]. During placentation, trophoblast cells are well-differentiated and acquire rapid proliferation and the ability to invade into the maternal endometrium to remodel utero-placental-arteries [34,35]. The tube formation is generally used for reflected placental angiogenesis and trophoblastic activity [36]. Our study demonstrated that a worse network formation was observed in α-solanine-treated HTR-8/SVneo cells, which may impair the differentiation of the extravillous trophoblast cells into endovascular trophoblasts. Aberrant trophoblast cells disturb the placentation, which causes serious complications during pregnancy, such as preeclampsia, recurrent pregnancy loss, fetal growth restriction, and abortion [37]. 

A single low dose of the major compounds of GAs derived in Solanaceae, including α-solanine, α-chaconine, α-tomatine, and solamargine, exerts toxic effects in many cancer cell lines [38]. The different routes of administration are closely related to GAs toxicity. The biotransformation of solamargine is quick and only a small amount of solamargine can be detected after 8 h of intravenous administration (4 mg/kg body weight) in rats [39]. solamargine is more toxic to embryos than solasonine [40]. The toxicity of α-tomatine is lower than other GAs; more than 40 mg/kg of α-tomatine caused the formation of abscesses via subcutaneous administration [41,42]. α-chaconine is the most toxic of the potato Gas; the lethal dose 50% was 27.5 mg/kg body weight through intraperitoneal injection in mice [43]. α-solanine, found at levels almost 100-fold higher in potato sprouts than tubers, has a toxicity similar to α-chaconine, cannot be removed through cooking, and easily accumulates in the body [44]. A combination of α-solanine and α-chaconine could synergistically induce the embryonic mortality and malformation in *Xenopus* [45]. In the present study, the cytotoxicity of high concentration of α-solanine on human trophoblast cells may impair placentation and affect the development and growth of fetus. Animal studies need to be done to confirm the adverse effect of α-solanine in placental development and its involvement in pregnancy outcome. 

Autophagy and apoptosis are two crucial and interconnected processes during placentation, which are often influenced by microenvironmental challenges [46,47]. Autophagy is a dynamic process and highly regulated with a dual role as pro-survival or pro-death. It physiologically participates in early normal gestation, and autophagic markers were abundant in trophoblast and decidual stromal cells [48,49]. α-solanine treatment promotes apoptosis in various cancer cells, such as liver, melanoma, and pancreatic cancer cells [4,7,9]. In line with these results, we also observed its apoptosis-promoting and autophagy-inducing effects in the human trophoblast cells. The ratio of LC3-II/LC3-I and the expression of Beclin 1 and Atg13 usually demonstrate the process of autophagy in cells [50,51]. In A549 cells, α-solanine exerts its cytotoxic effect though inducing autophagy and triggering apoptosis synergistically or in parallel [52]. α-solanine impairs the maturation of porcine oocyte by triggering autophagy as well as apoptosis, by increasing the levels of autophagy-related genes such as *LC3*, *LAMP2,* and *Atg7* and enhancing the accumulation of LC3-specific puncta [20]. Consistent with the above findings, we demonstrated that, when the HTR-8/SVneo cells were exposed to 20 µM α-solanine, Beclin 1 levels and the conversion of LC3-I to LC3-II (as ratio of LC3-II/LC3-I) peaked at 18 h. p62, an autophagic adaptor protein that binds both LC3 and polyubiquitinated proteins for degradation on autophagosomes, is negatively correlated with autophagy activation [53]. However, in the current study, p62 was not degraded with α-solanine-induced activation of autophagy, which may be due to the accumulation of dysfunctional proteins [54]. Autophagic inhibitor CQ can inhibit the fusion of lysosomes and autophagosome via raising the lysosomal pH [44]. Co-treatment with α-solanine and CQ increased the Beclin 1 protein level, the ratio of LC3-II/LC3-I, and yellow LC3 puncta in the human trophoblast cells, which may result from the accumulation of autophagosome in the cytoplasm induced by α-solanine. 

## 4. Conclusions

The present findings demonstrate that α-solanine exhibited its cytotoxic effect on human trophoblast cells. The inhibitory effect of α-solanine may be mediated by promoting autophagy and apoptosis in HTR-8/SVneo cells. More studies are needed to disclose the involvement of α-solanine in autophagy and apoptosis in trophoblast cells.

## 5. Materials and Methods 

### 5.1. Cell Culture

The immortalized human trophoblast cell line (HTR-8/SVneo) was provided by Dr. Tsang (Department of Obstetrics & Gynecology and Cellular & Molecular Medicine, University of Ottawa, Ottawa, ON, Canada). Cells were cultured with Dulbecco’s Modified Eagle Medium: Nutrient Mixture F12 (DMEM/F12) (Hyclone, Logan, UT, USA) containing 10% fetal bovine serum (FBS, Gibco Invitrogen Corporation, Carlsbad, CA, USA) and 1% penicillin–streptomycin (Beyotime Biotech, Nanjing, China) and incubated in a humidified incubator (Thermo Fisher Scientific, Waltham, MA, USA) with 5% CO_2_ at 37 °C.

### 5.2. Cell Proliferation Assay

Cell proliferation was assayed using CCK8 kits (Dojindo Molecular Technologies Inc., Shanghai, China). Cells were seeded in 96-well plates with 1 × 10^4^ cells per well. α-solanine (Sigma-Aldrich, St. Louis, MO, USA), dissolved in DMSO (Sigma-Aldrich), was diluted to the indicated concentrations with the basic culture medium. As a control, solvent without α-solanine was added to the cultures. When cells reached about 60% confluence, cells were incubated with different concentrations of α-solanine (0, 10, 20, 30, and 40 μM) for 24 h, six wells per dose. After treatment, 10 μL of CCK8 reagent was added and incubated for 3 h at 37 °C in the dark. The absorbance at 490 nm was read using a microplate reader (Thermo Fisher Scientific, USA). Three independent experiments were performed, and the data were normalized to the absorbance collected from the DMSO-treated cells.

### 5.3. Cell Cycle Assay

Cell cycle assay was performed using a Cell Cycles and Apoptosis Analysis Kit (Beyotime Biotech) according to the manufacturer’s instructions. Briefly, HTR-8/SVneo cells were seeded into 6-well plates, treated with α-solanine (0, 10, 20, and 30 μM) for 24 h. Cells were harvested and fixed in 70% ethanol overnight at 4 °C, and subsequently washed with phosphate buffered saline (PBS). PI solution containing 50 µg/mL RNase was then added to the cells and incubated at 37 °C for 30 min in the dark. Cell cycle analysis was performed on a Becton-Dickinson (BD) FACS system (Becton-Dickinson, San-Jose, CA, USA).

### 5.4. Cell Invasion and Migration Assays

Matrigel invasion and migration of HTR-8/SVneo cells were performed as described previously with modification [55]. Briefly, 5 × 10^5^ HTR-8/SVneo cells in 200 µL serum-free medium with or without α-solanine (0, 10, 20, and 30 μM) were seeded into the chamber of the transwell insert (pore size 8.0 µm, Corning Life Sciences, Acton, MA, USA), which was pre-coated with Matrigel (Becton Dickinson Biosciences, Franklin Lakes, NJ, USA). Medium containing 10% FBS was added to the 24-well plate. After culturing for 18 h, non-invaded cells were scraped off the upper surface of the insert with cotton swabs. The cells invading through the surface were fixed with 4% paraformaldehyde (PFA) for 20 min, and then stained with a 0.1% crystal violet solution for 30 min. Images of invaded cells from six random fields were photographed under a microscope (Olympus, IX73P1F, Tokyo, Japan) and counted using ImageJ software. Cell migration was detected using the procedures similar to those in cell invasion assay, except the upper chamber was not coated with Matrigel.

### 5.5. Tube Formation Assay

Networks formed in trophoblasts was evaluated utilizing tube formation assay as described previously with slight modifications [56]. Ninety-six-well plates were coated with Matrigel (50 μL per well) and solidified at 37 °C for 1 h. HTR-8/SVneo cells were suspended in serum-free DMEM/F12 medium containing different doses of α-solanine (0, 10, 20, and 30 μM) and seeded on the Matrigel at a density of 1 × 10^5^ cells per well. After incubating for 6 h, tube-like structures were observed under a microscope (Olympus, IX73P1F, Japan). The number of nodes, junctions, and tubules and the total branches length from five random fields were quantified by ImageJ software.

### 5.6. Cell Apoptosis Assay

An Annexin V-FITC/PI Apoptosis Detection kit (Thermo Fisher Scientific, USA) was used to stain apoptosis cells according the manufacturer’s instructions. After HTR-8/SVneo cells were treated with different concentrations of α-solanine (0, 10, 20, and 30 μM) for 18 h, cells were collected and washed once with cold PBS. Cells were resuspended with 100 μL of 1 × binding buffer followed by staining with Annexin V-FITC and PI solution. Then, 200 μL binding buffer was added. Cells were analyzed by flow cytometry using the software FlowJo-V10 (Tree Star, Ashland, OR, USA).

### 5.7. TUNEL Assay

TUNEL assay was performed using a DNA Fragmentation Detection Kit (Beyotime Biotech), as described previously [57]. Briefly, cells were treated with α-solanine for 18 h, fixed with 4% PFA for 30 min, rinsed with PBS, and permeabilized with Triton X-100 (0.1%) on ice. Then, cells were stained with TUNEL staining mixture for 1 h at 37 °C in the dark and counterstained with 4’, 6’diamidino-2-phenylindole (DAPI) for 5 min at room temperature. The apoptotic cells were observed using a fluorescence microscope (Olympus, IX73P1F, Japan). Cells from five random fields were counted using ImageJ software.

### 5.8. Western Blot

After treatment, cells were lysed in RIPA buffer (Beyotime) containing phenylmethylsulfonyl fluoride (1 mM, Sigma-Aldrich) for 30 min followed by centrifuge. Equal amounts of protein were separated by SDS-PAGE and transferred onto polyvinylidene difluoride (PVDF) membranes (Millipore Inc., USA). Membranes were blocked with 0.2% gelatin, then incubated with the indicated primary antibodies to Beclin 1 (ab62557, Abcam, Cambridge, UK), LC3 (ab51520, Abcam), Atg13 (GB11591, Servicebio, Wuhan, China), SQSTM1/p62 (GB11531, Servicebio) and GAPDH (KC-5G5, Kangcheng Bio-tech, Sichuan, China) at 4 °C overnight. After washing in TBST, the membranes were incubated with the corresponding horseradish peroxidase (HRP)-linked antibodies. The blots were developed using an enhanced chemiluminescence (ECL)-plus kit (Beyotime, China) and visualized by the ChemiDoc XRS+ system (Bio-Rad, Hercules, CA, USA).

### 5.9. Autophagic Flux

To monitor autophagic flux inducing by α-solanine, trophoblasts were transfected with a mCherry-GFP-LC3B vector (MiaoLingBio, Wuhan, China) via lentiviral transduction. After co-treatment with 20 μM α-solanine and 50 μM CQ or without for 18 h, cells were fixed with 4% PFA, and DAPI was used to dye nucleus. The fluorescence signals were checked using a fluorescence microscope (Olympus, BX53F, Japan).

### 5.10. Statistical Analysis

Results are presented as mean ± standard error (S.D.) from three independent experiments. Student’s T test or One-Way ANOVA test was used for statistical analysis. * *p* < 0.05, ** *p* < 0.01, and *** *p* < 0.001 were considered for statistical significance.

## Data Availability

Data are available upon request, please contact the contributing authors.

## Supplementary Materials

The following are available online at https://www.mdpi.com/2072-6651/13/1/67/s1, Figure S1: α-solanine induced the expression of autophagy-related biomarkers of HTR-8/SVneo cells.
