# Supplementary material for: α-Solanine Causes Cellular Dysfunction of Human Trophoblast Cells via Apoptosis and Autophagy
Source: Toxins (Basel). 2021 Jan 18;13(1):67. doi: 10.3390/toxins13010067 (PMC7830445; doi:10.3390/toxins13010067)
Supplement: Supplementary file 1 [file toxins-13-00067-s001.pdf]

Supplementary Materials: α-Solanine Causes Cellular Dysfunction of Human Trophoblast Cells via Apoptosis and Autophagy

Zhilong Chen, Chen Li, Anwen Yuan, Ting Gu, Feng Zhang, Xiujun Fan, Xiaosong Wu, Xingyao Xiong and Qing Yang

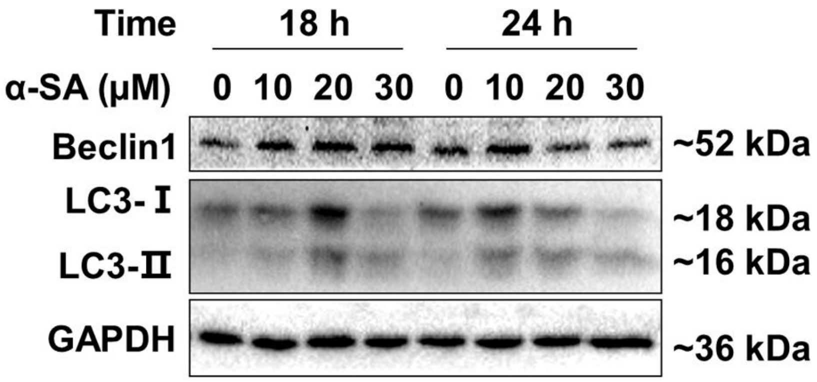

Figure S1. α-Solanine induced the expression of autophagy-related biomarkers of HTR-8/SVneo cells. Cells were exposed to α-Solanine (0, 10, 20 and 30 μM) for 18 h or 24 h, the expression of Belclin1 and LC3 were analyzed by western blot, GAPDH as a loading control.
